# Morbid Habit of Swallowing Hair; Prolonged Sojourn of the Foreign Bodies in the Gastro-intestinal Canal

**Published:** 1852-08

**Authors:** 


*Morbid Habit of Swallowing Hair; Prolonged sojourn of the foreign bodies in the gastro-intestinal canal. Evacuation of Packets of Hair by vomiting and alvine dejections.* (Under the care of Dr. Crawford, Middlesex Hospital.)—Dr. Thompson has alluded, in this journal, to the case of a girl who used to swallow her hair, and had lately vomited packets of it. The patient has since then, passed per anum, a large mass of the same organic product; this circumstance induced us to inquire more minutely into the case, and we learned from the girl the following facts.

She is a servant, twenty-three years of age, now pale and thin, but formerly ruddy and stout, and was admitted Nov. 16, 1851, under the care of Dr. Crawford, with very obstinate constipation. The patient began to menstruate at the age of twelve years, and at thirteen, while in a comfortable situation, contracted the habit of picking off her hair, biting, chewing, and at last swallowing it. She went on satisfying this depraved taste for four or five months, when, being reprimanded, she gave it up, and has never resumed the custom since.

Soon after this, the patient began to feel a pain under the false ribs, on the left side, just over the spleen and the large extremity of the stomach. She was treated in various ways, and at different hospitals and dispensaries, during several years, for this pain, no one, nor herself, suspecting that the above mentioned habit was the source of her malady. The general belief was, that she suffered from a tumour in the vicinity of the spleen; pain in that region, constipation of bowels, and wasting, being the principal symptoms.

At last, about a fortnight before admission, she was seized with fits of vomiting, and, among the rejected matters, a solid concretion, about the size of a walnut, was noticed; but this attracted no attention, until a second and much larger one was likewise brought up in the hospital. The nature of the affection became now apparent, but the constipation was very obstinate, and went so far as to produce stercoraceous vomiting. No more hair was noticed after these symptoms abated, until Jan. 26, about nine weeks after admission, when a very large hairy concretion was discovered in the fæces. It was of the size of the dilated rectum, measured five inches in length, and was of a deep black color. (The girl's hair is of a light tint.) The patient states that she felt this in the right iliac fossa, and she is now under the impression that more hair will be evacuated. The health has of late been rather weak, but the appetite is pretty good, and the intellect clear; but the patient complains of flatus, and of the bowels rolling in knots. This is another and very striking example of the difficulty of treating disease, when we do not know *every particular* of the history.—*London Lancet.*

